# Detection of NT-proBNP Using Optical Fiber Back-Reflection Plasmonic Biosensors

**DOI:** 10.3390/bios14040173

**Published:** 2024-04-04

**Authors:** Ana Sofia Assunção, Miguel Vidal, Maria João Martins, Ana Violeta Girão, Médéric Loyez, Christophe Caucheteur, José Mesquita-Bastos, Florinda M. Costa, Sónia O. Pereira, Cátia Leitão

**Affiliations:** 1 i3N, Department of Physics, University of Aveiro, 3810-193 Aveiro, Portugal; anasofia.matos@ua.pt (A.S.A.); miguelvidal@ua.pt (M.V.); mariajoao19@ua.pt (M.J.M.); flor@ua.pt (F.M.C.); 2 CICECO—Aveiro Institute of Materials, Department of Materials and Ceramics Engineering, University of Aveiro, 3810-193 Aveiro, Portugal; avgirao@ua.pt; 3 Electromagnetism and Telecommunication Department, University of Mons, 31 Bld Dolez, 7000 Mons, Belgium; mederic.loyez@umons.ac.be (M.L.); christophe.caucheteur@umons.ac.be (C.C.); 4 Institute of Biomedicine—iBiMED, School of Health Sciences, University of Aveiro, 3810-193 Aveiro, Portugal; mesquitabastos@gmail.com

**Keywords:** optical fiber uncladded tip biosensors, N-terminal B-type natriuretic peptide (NT-proBNP), surface plasmon resonance (SPR), gold plasmonics

## Abstract

Heart failure (HF) is a clinical entity included in cardiovascular diseases affecting millions of people worldwide, being a leading cause of hospitalization of older adults, and therefore imposing a substantial economic burden on healthcare systems. HF is characterized by dyspnea, fatigue, and edema associated with elevated blood levels of natriuretic peptides, such as N Terminal pro-B-type Natriuretic Peptide (NT-proBNP), for which there is a high demand for point of care testing (POCT) devices. Optical fiber (OF) biosensors offer a promising solution, capable of real-time detection, quantification, and monitoring of NT-proBNP concentrations in serum, saliva, or urine. In this study, immunosensors based on plasmonic uncladded OF tips were developed using OF with different core diameters (200 and 600 µm). The tips were characterized to bulk refractive index (RI), anddetection tests were conducted with NT-proBNP concentrations varying from 0.01 to 100 ng/mL. The 200 µm sensors showed an average total variation of 3.6 ± 2.5 mRIU, an average sensitivity of 50.5 mRIU/ng·mL^−1^, and a limit of detection (LOD) of 0.15 ng/mL, while the 600 µm sensors had a response of 6.1 ± 4.2 mRIU, a sensitivity of 102.8 mRIU/ng·mL^−1^, and an LOD of 0.11 ng/mL. Control tests were performed using interferents such as uric acid, glucose, and creatinine. The results show the potential of these sensors for their use in biological fluids.

## 1. Introduction

Cardiovascular diseases (CVDs) continue to be the leading cause of death worldwide, accounting for 32% of mortalities recorded annually, with an estimated 17.9 million deaths in 2019, which represents a serious public health problem [1]. Heart failure (HF) is the most disabling cardiovascular pathology, with the highest rates of morbidity and mortality, as well as the largest health-care expenses. It is identified by typical symptoms, such as dyspnea, lower limb edema, and fatigue, which may be accompanied by alterations in cardiac structure or function, resulting in decreased cardiac output and/or increased intracardiac pressures at rest or during exercise. Dyspnea and weariness, the most prevalent symptoms, have a considerable detrimental impact on functional ability and quality of life [2,3].

The high costs of HF can be attributed to various factors, namely hospitalizations, constant readmissions and loss of productivity [1,4]. It is important to note that more than 50% of patients with HF end up dying within 5 years after the first hospitalization, which reveals a lower survival rate compared to cancers, such as breast, prostate, bladder, and colorectal [5].

To overcome diagnosis limitations and enhance HF risk stratification, attempts were undertaken to study the disease’s pathophysiology at the cellular level, namely the molecules that end up being secreted as a regulatory mechanism for HF. Biomarkers are biological molecules that can be found in physiological fluids such as blood, urine, and saliva [6]. Natriuretic peptides, particularly B-type Natriuretic Peptide (BNP) and its N-Terminal portion (NT-proBNP), are well-established cardiovascular biomarkers for HF and are regarded as the gold standard, due to their relevance and specificity to this health condition. The European Society of Cardiology (ESC) 2021 guidelines set that outpatients with NT-proBNP plasma concentration > 125 pg/mL are diagnosed with chronic HF. Higher values, >300 pg/mL, are suggestive of acute HF. So, NT-proBNP concentrations below these values have a substantial negative predictive value for HF [7,8]. Laboratory tests based on immunoassays are currently employed for illness detection and exclusion [4].

Enzyme-Linked Immunosorbent Assay (ELISA) and Polymerase Chain Reaction (PCR) are two conventional methods for quantification of HF biomarkers. These are time consuming (2 to 8 h) techniques that require advanced and expensive equipment that needs to be handled by experienced technicians and situated in centralized laboratories [9]. Therefore, researchers are motivated in developing new biosensing tools to be used for Point-Of-Care Testing (POCT). These tests must be cost-effective, compact, portable, and user-friendly, as well as capable of providing accurate and real-time bedside monitoring, which is critical in HF diagnosis and progress assessment [10]. In this context, efforts have been focused on the development of optical biosensors for the detection and quantification of NT-proBNP biomarkers, owing to their fast response times and high sensitivity [4].

The surface plasmon resonance (SPR) effect is a promising optical method that occurs when electromagnetic waves, traveling in a dielectric medium, contact with specific metallic thin coatings by total internal reflection, causing surface plasmon polaritons to propagate along the metal–dielectric interface. This approach is extremely useful in biological applications because SPR conditions are extremely sensitive to changes in the refractive index (RI) at the transducer surface (metal–dielectric contact). Therefore, biomolecular interactions between the target analyte and the immobilized biological receptor at the transducer will change the surface RI, consequently inducing spectral changes of the reflected light. Thus, an SPR-based biosensor has numerous advantages, such as allowing the detection of a biomolecular interaction without the need for markers (label-free approach), real-time observation of the binding, and determination of concentrations of the analyte of interest [11].

In recent years, optical fibers (OFs) have demonstrated great potential as substitute substrates for the classic prisms commonly employed in SPR technology, namely due to the fact of being easy to handle, light in weight, inexpensive, immune to electromagnetic interference, and resistant to harsh conditions. For an OF to be used as plasmonic substrate, except for diffraction gratings, its geometry must be altered for better coupling between the guided modes of core and the surface plasmons, enhancing the sensitivity of the sensor to changes in the surrounding medium [12]. In general, the geometrically modified portion of the OF is coated with a metal layer, which is surrounded by a dielectric layer (usually the medium being monitored). The light injected into the OF, typically polychromatic, is propagated by total internal reflection and the consequent evanescent wave excites the surface plasmons at the metal–fiber interface. This coupling depends on the wavelength of the light, OF features, geometry, and metal properties [13].

In the case of this work, gold-coated OF tips are tested for the detection of NT-proBNP, comparing two different core diameters: 200 and 600 μm. This sensing approach was selected due to the small dimensions, high RI sensitivity, and possibility to be easily immersed in the detection medium [14].

## 2. Materials and Methods

### 2.1. Reagents

The following reagents were acquired and used as received: glucose (D-(+)-Glucose ≥99.5%, GC), cysteamine (Cys) hydrochloride (≥98%), *N*-(3-dimethylaminopropyl)-*N′*-ethylcarbodiimide hydrochloride (EDC), and *N*-hydroxysuccinimide (NHS), from Merck, Germany; phosphate-buffered saline (PBS) tablets (pH = 7.4, 10 mM), from Fisher Bioreagents (Pittsburgh, PA, USA); bovine serum albumin (BSA), from Alfa Aesar (Haverhill, MA, USA); anti-NT-proBNP antibodies (ABs) and NT-proBNP peptide, from antibodies-online GmbH, (Aachen, Germany). Throughout the work, deionized (DI) water was used that was obtained from a Milli-Q water purification system.

### 2.2. Plasmonic OF Tips Production

The sensors were manufactured with step-index multimode plastic cladding fibers with silica cores of 200 (FP200URT) and 600 μm (FP600URT), from Thorlabs (Newton, NJ, USA). These sensors will be called 200 µm tips and 600 µm tips, respectively. Pieces with 15 cm of fiber were cut and 3 cm of external coating was stripped with a blade at both ends. On one end, the cladding was gently removed with a blade and then wiped with a cloth soaked in ethanol to remove the cladding remnants. The 200 µm tips were then cleaved with an optical fiber cleaver (Model FC-6S, Sumitomo Electric Industrial Co. Ltd., Tokyo, Japan) to obtain 1 cm of core exposition for further coating with gold. In the case of the 600 µm tips, a polishing sheet was used to ensure a smooth surface tip at both ends. The other end of the fibers was also cleaved to connect the fibers to the interrogation setup. The cleaving quality was evaluated using an optical microscope (Swift M10L, Swift Optical Instruments, San Antonio, TX, USA).

Afterwards, a gold nanocoating was deposited on the 1 cm unclad tip of each fiber, using a sputter coater (Polaron SEM coating unit E5100, Quorum Technologies Ltd., Ringmer, UK). Batches of 200 µm tips and 600 µm tips were placed inside the chamber aligned with the gold target. The distance between the OFs and the target was about 5 cm and the coating process was performed inside the chamber at a pressure of about 10^−2^ torr, a current of 10.25 mA, and a voltage of 1.2 kV. The fibers were rotated 180° to ensure complete coating of the surface of the sensing region. The coating duration was 80 s for 200 µm tips and 120 s for 600 µm tips, on each side, since the latter has a larger contact surface area. The opposite ends of the fibers were covered to prevent coating.

**Figure 1:**
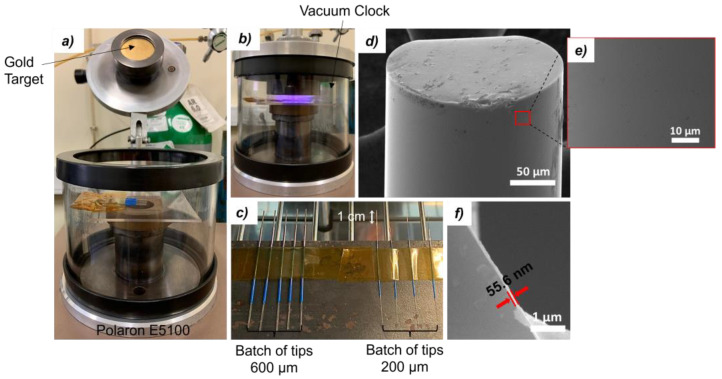
Pictures of (**a**) the gold sputter coater, where OF tips are placed horizontally inside the vacuum chamber, (**b**) the sputtering process, (**c**) the batches of 200 µm tips and 600 µm tips, ready to be annealed, and (**d**–**f**) SEM images of an OF tip after thermal annealing.

Figure 1a and Figure 1b depict the coating chamber and process, respectively. The coated tips, shown in Figure 1c, were then annealed in a thermal chamber, at 180 °C for 2 h, to enhance the adhesion of the gold film to the silica surface, also improving its conductivity by better aggregation of its grains. [15]. Figure 1d,e show the surface of a tip after thermal annealing. The tips before annealing are displayed in Figures S3 and S4, detailed in the Supplementary Materials. Finally, in Figure 1f, the thickness of the gold layer after annealing was measured, being around 56 nm (See Figure S4, Supplementary Materials). Indeed, full morphological and chemical characterization was performed for OF tips throughout the various stages of the immunosensor development, as presented in the Supplementary Materials.

### 2.3. Experimental Setup and Data Analysis

**Figure 2:**
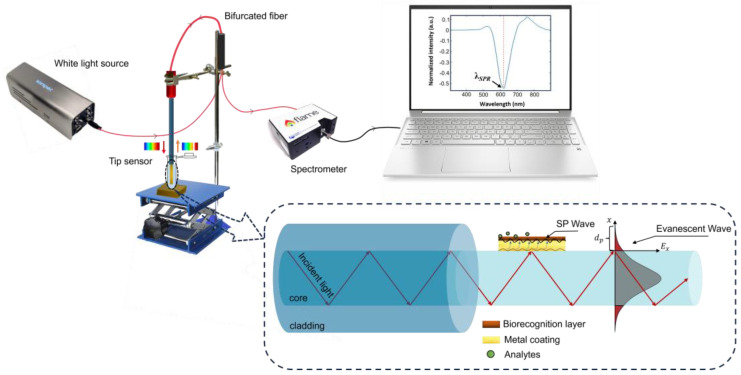
Schematic diagram of the experimental set-up for a gold coated OF tip (image not to scale).

The OF tips were accessed using the experimental setup presented in Figure 2. The OF tip is introduced at the bare fiber terminator (BFT1, Thorlabs), connected to an optical fiber 1 × 2 coupler (FCB-UVIR200-1 or FCB-UVIR600-1, Avantes, Apeldoorn, The Netherlands), which on the other hand is linked to a light source (LS-W7, Sarspec, Vila Nova de Gaia, Portugal), emitting between 380 and 2500 nm, and a spectrometer (FLAME-T-UV–vis, Ocean Optics, Orlando, FL, USA), to monitor the reflected light in the spectral range of 380 to 890 nm (0.19 nm resolution). This setup allows real-time monitoring of the reflected spectra and data acquisition by the OceanView 2.0.8 software (Ocean Optics, Orlando, FL, USA). A 250 µL Eppendorf safe-lock tube was used as a container to perform the RI characterization, the functionalization procedures, and the detection tests. It was fixed to a lab jack elevator allowing solution replacement without OF tip manipulation.

The reflected light by the OF tip, traveling backwards, includes all wavelengths injected into the fiber except for the band absorbed by the gold, centered at the resonance wavelength, *λ_SPR_*. The reflectivity spectrum is obtained by the difference between the normalized spectrum acquired in the test solution and the reference spectrum in air. A Fast Fourier Transform filtering method with 40 points of window was applied to remove the high-frequency noise. Then, to study the optical response of the tips, the minimum reflectivity (*λ_SPR_*) was identified and plotted as a function of each evaluated variable (RI and NT-proBNP concentration). All data processing was carried out this way, using Origin software (OriginLab, Northampton, MA, USA).

### 2.4. Characterization to Bulk RI

The produced 200 and 600 µm tips were all characterized to bulk RI and the sensitivity of each one was obtained. The RI characterization was carried out using DI water, followed by glucose solutions of increased concentrations: 0.1, 0.5, 1, 5, 10, 20, 30, 40, and 50% (*w*/*v*). A refractometer (Abbemat 200, Anton Paar, Graz, Austria) was used to determine the respective RI values: 1.3332, 1.3336, 1.3342, 1.3396, 1.3461, 1.3566, 1.3674, 1.3765, and 1.3853 RIU. Tip cleaning, before and after the characterization process, involved sequential steps: (1) pure ethanol (twice, 2 min each), (2) 10% (*v*/*v*) aqueous ethanol solution (once, 2 min), and (3) DI water (twice, 2 min each).

For each tip, the *λ_SPR_* (nm) was plotted as a function of RI. Then, the RI sensitivity was determined by the first derivative of the fit that best modulated the data and expressed as wavelength shift per RI units variation (nm/RIU).

### 2.5. Biofunctionalization

**Figure 3:**
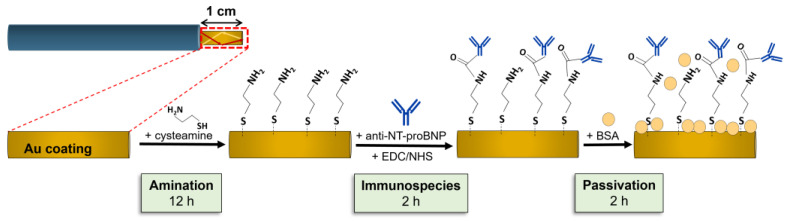
Plasmonic OF tip and respective biofunctionalization steps for biosensor fabrication.

The tips with highest bulk RI sensitivity were biofunctionalized, adapting the procedure from elsewhere [16]. The process started by immersing each tip in 250 μL of a 200 mM aqueous Cys solution. The fiber’s sensing region remained in this solution overnight, creating a Cys self-assembled monolayer. After overnight incubation, the fibers were washed thrice with DI water and then PBS to remove unbound Cys. After this first step, an optical spectrum was acquired. Following this, anti-NT-proBNP ABs were covalently linked to the aminated surface. For this, a mixture of 100 μL anti-NT-proBNP ABs (500 μg/mL), 50 μL EDC (0.1 M), and 50 μL NHS (0.2 M), both in PBS, was prepared. The tip was immersed in this mixture of 200 µL for 2 h. At the end, the sensor underwent three PBS washes, and an optical spectrum was recorded. To finalize, the tip surface was passivated using 250 μL of BSA solution (10 μg/mL in PBS) for 2 h, to minimize non-specific links. After PBS washing, a new optical spectrum was recorded. The biofunctionalization procedure’s steps are schematized in Figure 3 for clarity.

### 2.6. NT-proBNP Detection

The immunosensors were tested using different NT-proBNP concentrations. Each biofunctionalized tip was immersed in 5 solutions of 250 μL NT-proBNP in PBS, at a concentration range of 0.01, 0.1, 1, 10, and 100 ng/mL. The study of this wide range of concentrations allows for the whole response of each immunosensor, including in the range of biological interest, where the cut-off values are 0.125 ng/mL or 0.300 ng/mL for the diagnosis of chronic or acute HF, respectively.

Initially, a 30 min immersion in PBS served as the baseline (0 ng/mL peptide concentration), also assuring that the OF-tip response is stable. Then, each tip sensor was successively immersed in NT-proBNP solutions, from 0.01 to 100 ng/mL. A 30 min incubation per solution ensured AB–peptide complex formation and resonance wavelength stabilization. After each immersion, a PBS cleaning step removed unbound peptides. Subsequently, spectral acquisition was performed in PBS. Immunosensors’ performance was evaluated and compared based on fiber core diameter.

### 2.7. Control Tests

A control test was carried out using uric acid, glucose, and creatinine as interferent molecules, with the goal of evaluating the specificity of the immunosensors produced in terms of affinity with the specific anti-NT-proBNP ABs. These interferents, present in blood, urine, and saliva, were chosen due to their physiological relevance. Approximate mean values for these interferents in blood were used: 4 mg/dL uric acid, 80 mg/dL glucose, and 0.8 mg/dL creatinine. For this procedure, fibers were biofunctionalized as outlined in Section 2.4 with Cys, anti-NT-proBNP ABs, and BSA. After a 30 min PBS incubation, each sensor was immersed for 30 min in PBS solutions of interferents (0.04 mg/mL uric acid, 0.8 mg/mL glucose, and 8 μg/mL creatinine) following a PBS washing step and spectral acquisition. Subsequently, sensors were tested to 5 different NT-proBNP concentrations. Each sensor underwent a 30 min immersion in 250 μL of each NT-proBNP solution, in increasing order of concentration. PBS washing step and optical spectrum acquisition occurred after each concentration.

## 3. Results and Discussion

### 3.1. Characterization to RI

**Figure 4:**
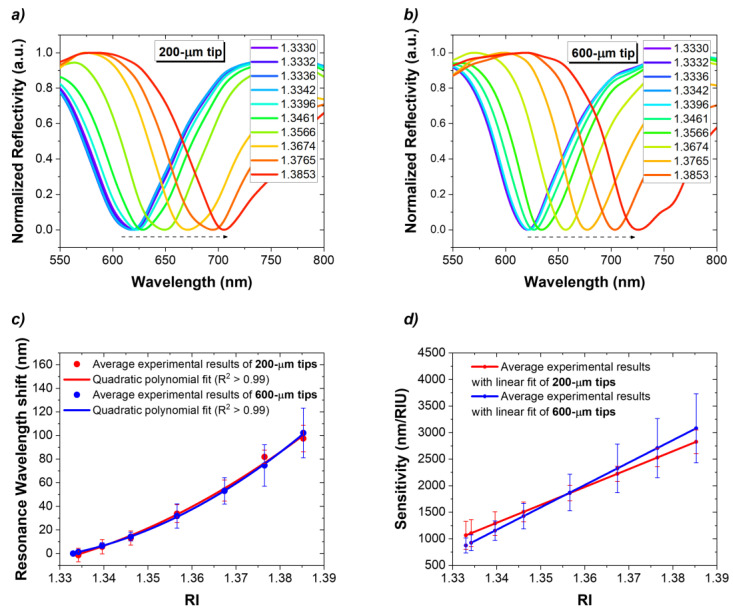
Characterization to RI: reflectivity spectra recorded in solutions with RI ranging from 1.3330 to 1.3853 RIU for (**a**) a 200 µm tip and (**b**) a 600 µm tip; (**c**) *λ_SPR_* shift as a function of RI with respective polynomial fits, Δ*λ_SPR_* = 18,131.3RI^2^ − 47,552.7RI + 31,783.3 and Δ*λ_SPR_* = 22,346.7RI^2^ − 58,810.3RI + 39,293.1 for the 200 µm tips (n = 13) and 600 µm tips (n = 13), respectively; (**d**) RI sensitivities expressed as *S* = 36,262.6RI – 47,552.7 and *S* = 44,693.3RI − 58,810.3 for the 200 µm tips and 600 µm tips, respectively.

To study the RI response reproducibility of the fiber tips of both diameters, several sensors were manufactured for each diameter and characterized using various aqueous glucose solutions with specific RIs. In all sensors, spectra analysis reveals that the position of SPR signatures undergoes a redshift with increasing RI values. As a matter of example, Figure 4a,b show the results for a 200 µm tip (Δ*λ_SPR_* = 88.7 nm) and a 600 µm tip (Δ*λ_SPR_* = 101.9 nm), respectively. A total of 26 OF tips (13 tips of each diameter) were characterized to RI. The response of the 200 µm tips and 600 µm tips is presented in Figure 4. Furthermore, the baseline *λ_SPR_* of both OF tips is very similar (612.2 nm for 200 µm tips and 606.5 nm for 600 µm tips), which reenforces that the gold thickness of the fibers is similar. The results show that a second order polynomial curve is the best fit for modelling the data, indicating that the RI dependence is nonlinear, showing smaller *λ_SPR_* for lower RIs.

To characterize the response to RI variation, the sensitivity (*S*) was expressed by Equation (1) as the change in resonance wavelength (*δλ_SPR_*) per unit change in RI (*δRI*) of the sensing medium.

(1) $$S=\frac{\delta \lambda_{SPR}}{\delta RI}\left[\frac{nm}{RIU}\right]$$

The sensitivity was determined from the first derivative of the quadratic fit equation used in the characterization of each type of tip, as shown in Figure 4d. As expected, *S* increased with RI. For 200 µm tips, a sensitivity was determined and increased from 1065.7 to 2826.8 nm/RIU along this RI range. In the case of 600 µm tips, the fibers’ sensitivity varies between 874.5 and 3080.7 nm/RIU. It can be verified that the 200 µm tips reached a higher sensitivity in the lower RI range, 1065.7 vs. 874.5 nm/RIU attained for the 600 µm tips, which in turn presented an average higher sensitivity for the higher RIs, 3080.7 vs. 2826.8 nm/RIU for the 200 µm tips.

### 3.2. Biofunctionalization

**Figure 5:**
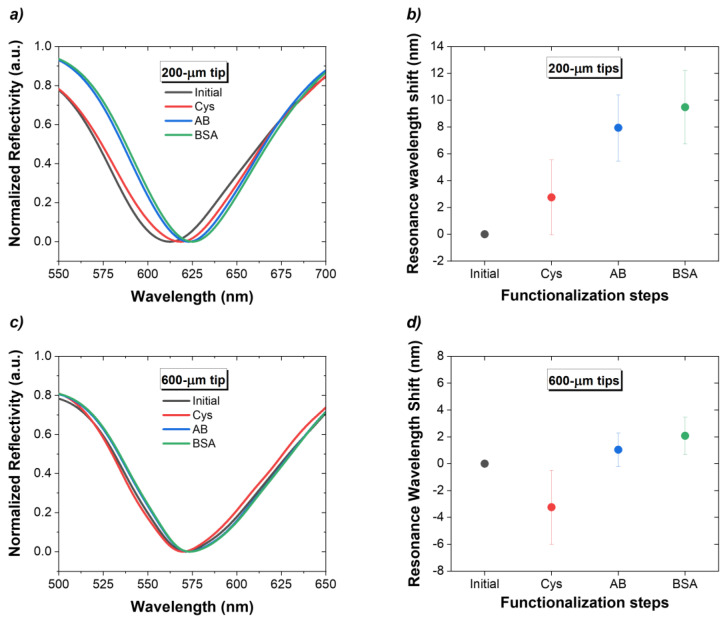
Tips response during biofunctionalization: reflection spectra acquired in PBS after each functionalization step with respective Δ*λ_SPR_* for (**a**,**b**) 200 µm tips and (**c**,**d**) 600 µm tips.

Figure 5a,c show examples of the spectra acquired during each biofunctionalization step. As explained before, the label-free detection of the biochemical reactions that occur on the fiber surface is performed by monitoring the RI variations. Therefore, it is important to acquire all optical spectra in PBS after each functionalization step, to guarantee that the Δ*λ_SPR_* are due to the molecules bound to the surface. Figure 5b,d show the average Δ*λ_SPR_*, caused by each of the four steps of the biofunctionalization procedure for n = 3. At the end of this process, there was an average redshift of the total SPR wavelength of 9.5 ± 2.8 nm for 200 µm tips and of 2.1 ± 1.4 nm for 600 µm tips.

### 3.3. NT-proBNP Detection

**Figure 6:**
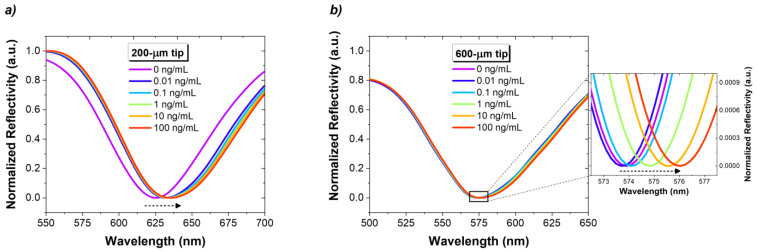
Reflectivity spectra, acquired in PBS, after 30 min incubation periods in NT-proBNP solutions in a concentration range of 0.01–100 ng/mL: (**a**) 200 µm tip and (**b**) 600 µm tip.

The immunosensors reported in the last section were tested for different concentrations of NT-proBNP, and the respective reflection spectra were acquired. Figure 6 shows the spectral response of a 200 µm tip and a 600 µm tip, for increasing concentrations of NT-proBNP from 0.01 to 100 ng/mL.

**Figure 7:**
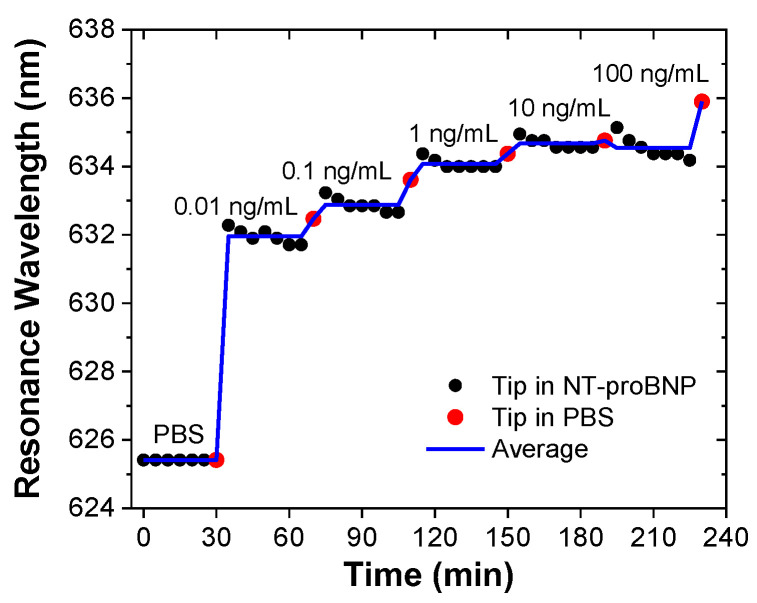
Example of a tip sensor response over time, exposing a 200 µm tip biosensor to 5 different concentrations of NT-proBNP, in steps of 30 min, for a total of 230 min (the chart displays the raw data in dots, with the black dots being the acquisitions in NT-proBNP solutions and the red dots in PBS solution after the washing step, and the average trace in blue).

As an example, Figure 7 shows the response over time of one of the immunosensors, where it is possible to observe the wavelength redshift with increasing concentrations of the biomarker.

**Figure 8:**
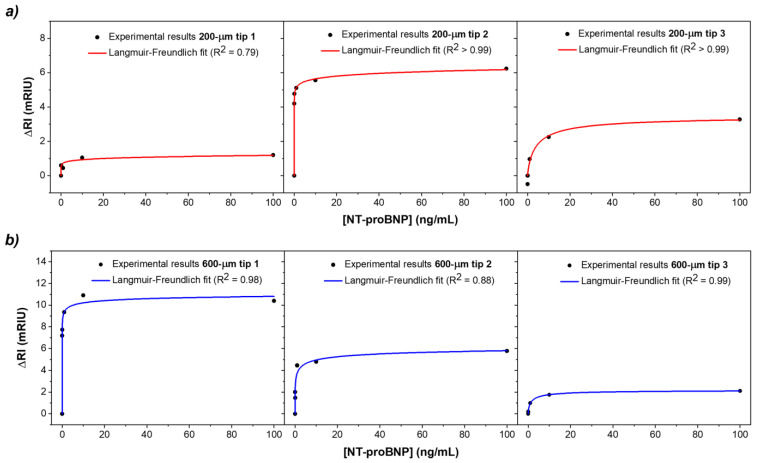
Tips response in ∆RI of (**a**) 200 µm tips (n = 3) and (**b**) 600 µm tips (n = 3), exposed to NT-proBNP concentrations ranging from 0.01 ng/mL to 100 ng/mL, showing the Langmuir–Freundlich fit to the experimental data.

To compare the biosensors’ performance during detection, the fitting curves presented in Section 3.1 for the three tip batches were considered and the response in *λ_SPR_* was translated to RI as a function of the NT-proBNP concentration. The results for both biosensors’ batches are presented in Figure 8.

Regarding the ability to detect the target peptide, both types of sensors show a similar trend with increasing concentration of NT-proBNP from 0.01 to 100 ng/mL. The concentration dependence may be explained with the Langmuir–Freundlich isotherm, as shown by the adequate fitting of the data in Figure 8. This is a mathematical model that includes the interaction between analyte and ligand binding sites and thus accounts for the saturation effect, which can be expressed by Equation (2):

(2) $$∆RI={∆RI}_{max}\frac{{\left(kc\right)}^{n}}{1+{\left(kc\right)}^{n}}\left[\frac{mRIU}{ng\cdot {mL}^{-1}}\right],$$

where Δ*RI* is the immunosensor response (in RI change) as a function of the analyte concentration, *c*; Δ*RI_max_* refers the maximum response value at saturation; *k* indicates the affinity between anti-NT-proBNP antibodies and NT-proBNP antigens; and *n* is the index of heterogeneity [17].

It is shown that the limited number of receptor sites at the gold–dielectric interface ends up saturating for high concentrations of NT-proBNP (>10 ng/mL), which indicates that all binding sites are filled. The average response of the 200 µm tips showed an *R*^2^ = 0.97, while for the 600 µm tips, an *R*^2^ = 0.96 was obtained. It can be verified that the 600 µm tips present higher sensitivity for NT-proBNP detection, exhibiting an average ΔRI of 6.1 ± 4.2 mRIU compared to 3.6 ± 2.5 mRIU for the 200 µm tips. Indeed, the larger core size of the fibers is responsible for the improvement in the sensitivity of the biosensor. In this regard, it is possible to determine the sensitivity defined as the slope of the linear regression obtained for the RI variation as a function of NT-proBNP concentration, in the initial region of the response. The 200 µm tips showed an average sensitivity of 50.5 mRIU/ng·mL^−1^, while the 600 µm tips exhibited a value of 102.8 mRIU/ng·mL^−1^. Besides the higher sensitivity, better reproducibility than the 200 µm tips was verified. One of the reasons for the higher reproducibility may be the fact that the 600 µm tips tested belonged to the same batch of fibers, i.e., they were subjected to the gold deposition and annealing processes at the same time.

The performance of the immunosensors was also assessed and compared based on the limit of detection (*LOD*). This figure of merit was determined using the IUPAC method, shown in Equation (3).

(3) $$LOD=\frac{3\sigma }{S}\;\left[\;\frac{ng}{mL}\;\right],$$

where σ is the average standard deviation of n = 3 tips taken from the signal obtained from the minimum analyte concentration of 0.01 ng/mL and *S* corresponding to the sensitivity calculated above from the slope of the linear regression. The LOD values for 200 µm tips and 600 µm tips were calculated using Equation (3) from the linear regions of the ΔRI as a function of analyte concentration, which was verified for the smaller concentrations, in the 0.001–0.01 ng/mL range. For the 200 µm tips, an LOD of 0.15 ng/mL was obtained, whereas for the 600 µm tips, an LOD of 0.11 ng/mL was achieved.

### 3.4. Control Tests

**Figure 9:**
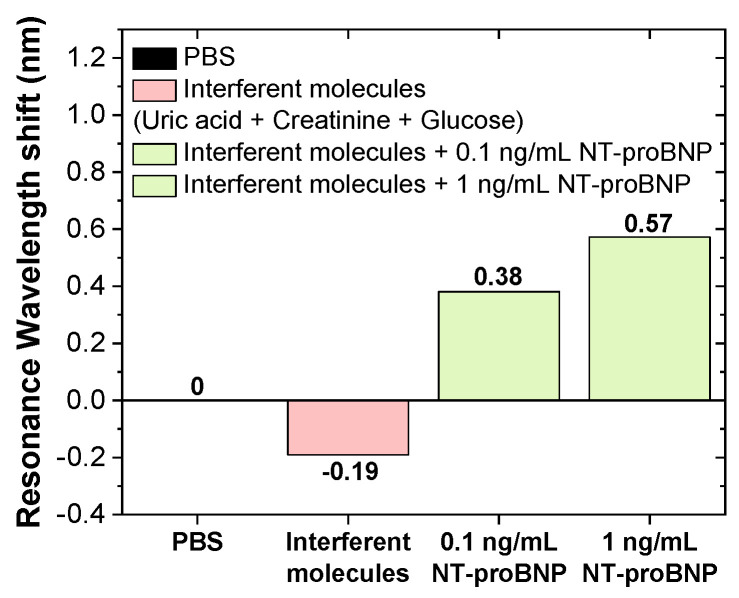
Bar graph comparison of the shift in resonance wavelength for the negative control (solution of glucose, acid uric, and creatinine) and two positive controls with different concentrations of NT-proBNP diluted in the interferent molecules’ solution, for a 600 µm tip.

After showing that the developed biosensors responded to the target of interest, their specificity was evaluated. for its assessment, the sensor surface was modified with anti-NT-proBNP ABs, as previously described, and subsequently incubated in the negative control (interferents molecules diluted in PBS) followed by positive controls (NT-proBNP with concentrations of 0.1 and 1 ng/mL diluted in the solution with interferent molecules). The resonance wavelength shift for these controls is shown as a bar graph in Figure 9.

The results show a concentration-dependent trend of NT-proBNP in the solutions that contained the specific analyte along with the other interferent compounds in the solution. These results showed the potential of the biosensor for the specific recognition of NT-proBNP, suggesting future studies for detection capability in samples of human fluids, such as blood.

### 3.5. Critical Analysis, Comparison of Tips, and Contribution to the State of the Art

The performance of 200 µm tips was compared with the 600 µm tips to understand which OF sensing approach would be the most advantageous for future HF biosensors. Starting with the manufacturing process, it is a quick process; however, the manual cutting and polishing processes could make it less reproducible due to human errors, since the OF tips may not always be cleaved and polished in the same way and free of irregularities. Extra care must be taken to ensure that the surface is as clean as possible, avoiding cladding residue, as this can compromise the gold coating uniformity, decreasing the efficiency of the light coupling between the fiber and the gold, and consequently the sensitivity to RI. Nevertheless, this is not a limitation for industrial production, as these processes can be easily automated to guarantee a high level of repeatability.

The gold sputtering method allows depositing gold directly on the bare glass surface of the tip with good adhesion, avoiding additional adhesion protocols. A uniform gold film over the sensing region should be assured, with a gold thickness around 50 nm essential for the highest sensitivity [18,19]. The double deposition technique requires greater control over deposition time and target substrate distance and can cause differential deposition due to variations in the angle of incidence of sputtering gold atoms on curved surfaces. Therefore, different production batches can result in slightly different RI sensitivities. It was concluded that the 200 µm tips presented higher sensitivity for lower RIs, while the 600 µm tips for the higher RIs, which resulted in a higher Δ*λ_SPR_* for the latter during detection of NT-proBNP.

The different types of biosensors developed to date for the detection of the cardiac biomarker NT-proBNP are summarized in Table 1. There is an increase in studies based on OFs due to the ease of handling and rapid analysis of results, compared to other immunosensors. Even though two studies have been already carried out with OF SPR sensors [20,21], the present work advances OF plasmonic sensors in the detection of this biomarker, as it tests a reflection configuration with low-cost instrumentation and interrogation at the visible wavelengths. The fact that it is a tip also simplifies the characterization and detection process as it can be used with a dip probe, particularly for submerging in body fluids or even in in vivo applications.

## 4. Conclusions

This work focuses on developing OF immunosensors based on SPR for the detection of an HF biomarker, NT-proBNP. Two types of OF sensors with a tip configuration were manufactured, tested, and experimentally compared: one with a 200 μm core diameter and the other with a 600 μm core diameter. The fibers were modified by removing the cladding of the fiber tip and coated with a gold film by the sputtering process. Thermal annealing was performed to even out the surface and enhance the SPR effect. Optical characterization was conducted to determine their sensitivity to RI. This parameter was obtained by monitoring the resonance wavelength of the spectrum obtained in each tested solution, where it was possible to determine an experimental average sensitivity between 1065.7 and 2826.8 nm/RIU for the 200 µm tips, and 874.5–3080.7 nm/RIU for the 600 µm tips. The biofunctionalization process was performed on six sensors (three 200 µm tips and three 600 µm tips), and the results showed that both biosensor types responded by a Langmuir–Freundlich fit to the presence of an NT-proBNP biomarker. The ΔRI varied to higher values with increasing NT-proBNP concentration. By presenting the ΔRI as a function of the Langmuir–Freundlich of the NT-proBNP concentration, it was possible to perform an initial linear fitting, obtaining an average sensitivity of 50.5 mRIU/ng·mL^−1^ and an LOD of 0.15 ng/mL for the 200 µm tips, while for the 600 µm tips, a higher sensitivity of 102.8 mRIU/ng·mL^−1^ and an LOD of 0.11 ng/mL were achieved. The detection method reported in this work provides a simple way to detect an important HF biomarker, which may be relevant in the evolving at-home, personal medicine environment.

The proposed SPR-based fiber optic biosensor offers a promising new way to detect an important cardiac biomarker, providing key elements for the future development of a rapid HF diagnostic and prognostic biosensor.

## Figures and Tables

**Table 1:** Summary of performance parameters of different biosensors for detection of NT-proBNP.

Methodology	Test Medium	Detection Range (ng/mL)	LOD (ng/mL)	Reference
Electrochemiluminescence	Human plasma	1 × 10^−5^–0.1	3.9 × 10^−6^	[22]
Gold electrode	Artificial Human saliva	[1–20] × 10^−3^	-	[23]
Field-effect transistor-based immunosensor	Human saliva	[2–100] × 10^−5^	2 × 10^−5^	[24]
Planar SPR	Porcine plasma	0.5–10	1.3	[25]
SPR excessively Tilted Fiber Grating	Human serum	0–1	0.5	[20]
SPR Tilted Fiber Bragg Grating	PBS	0.01–100	0.19	[21]
LSPR-Based Aptasensor	Human urine	0.6–6.0	0.417	[26]
OF nanogold-linked immunosorbent assay	Human plasma	5 × 10^−4^–5	5.8 × 10^−5^	[27]
SPR OF tips	PBS	0.01–100	0.15 (200 µm) 0.11 (600 µm)	This work

## Data Availability

The data of this paper are available upon request to the corresponding author.

## Supplementary Materials

The following supporting information can be downloaded at: https://www.mdpi.com/article/10.3390/bios14040173/s1, Figure S1. (a) SEM image and (b) EDS mapping of an uncladded fiber tip, Figure S2. (a) SEM image and (b) EDS mapping of the cross-section of a gold-coated tip containing remanent cladding around the core, Figure S3. SEM images of a gold-coated 200 µm tip before the thermal annealing treatment, Figure S4. SEM images of a gold-coated 200 µm tip after the thermal annealing treatment. In (c), the detail of the gold-film thickness is observed, Figure S5. (a) SEM image and (b) EDS mapping of gold-coated uncladded tip, Figure S6. (a) SEM image and (b) EDS mapping of a tip after the biofunctionalization and NT-proBNP detection tests, Figure S7. Contact-mode AFM 3D topography images made on (a) bare and (b) biofunctionalized 200 µm tips. Scan areas: 1 µm^2^.
