# Supplementary material for: Detection of NT-proBNP Using Optical Fiber Back-Reflection Plasmonic Biosensors
Source: Biosensors (Basel). 2024 Apr 4;14(4):173. doi: 10.3390/bios14040173 (PMC11048293; doi:10.3390/bios14040173)
Supplement: Supplementary file 1 [file biosensors-14-00173-s001.zip › biosensors-2880044-supplementary.pdf]

Supplementary Material

Detection of NT-proBNP Using Optical Fiber Back-Reflection Plasmonic Biosensors

Ana Sofia Assunção[1], Miguel Vidal[1], Maria João Martins[1], Ana Violeta Girão[2], Médéric Loyez[3], Christophe Caucheteur[3], José Mesquita-Bastos[4], Florinda M. Costa[1], Sónia O. Pereira[1,*] and Cátia Leitão[1,*]

[1] i3N, Department of Physics, University of Aveiro, 3810-193 Aveiro, Portugal; anasofia.matos@ua.pt (A.S.A.); miguelvidal@ua.pt (M.V.); mariajoao19@ua.pt (M.J.M.); flor@ua.pt (F.M.C.)
[2] CICECO—Aveiro Institute of Materials, Department of Materials and Ceramics Engineering, University of Aveiro, 3810-193 Aveiro, Portugal; avgirao@ua.pt (A.V.G.)
[3] Electromagnetism and Telecommunication Department, University of Mons, 31 Bld Dolez, 7000 Mons, Belgium; mederic.loyez@umons.ac.be (M.L.); christophe.caucheteur@umons.ac.be (C.C.)
[4] Institute of Biomedicine—iBiMED, School of Health Sciences, University of Aveiro, 3810-193 Aveiro, Portugal; mesquitabastos@gmail.com
* Correspondence: sonia.pereira@ua.pt (S.O.P.); catia.leitao@ua.pt (C.L.)

Contents

S1. Morphologic and Structural Characterization 1
S1.1 Preparation of OF tip 1
S1.2 Gold coating on OF tip 2
S1.3 Biofunctionalization of a gold-coated OF tip 3

S1. Morphologic and Structural Characterization

Optical fiber tips with 200 μm core diameter were subjected to a morphologic and structural characterization throughout the several steps for the immunosensor development.

SEM-EDX was employed to track surface modifications of the surface of OF 200 μm tips during immunosensor preparation stages. For that, the FEG-SEM Hitachi SU70 microscope was used, operating at 10.0 kV, equipped with an EDS Bruker. The OF tips were attached to an aluminum sample holder with double-sided carbon tape.

The surface roughness of biofunctionalized fibers was assessed through AFM images, comparing an annealed and non-functionalized 200 μm tip with a functionalized one. For this characterization, an Atomic Force Microscope from Bruker (Veeco), model Multimode Nanoscope Instruments IIIA, was used.

S1.1 Preparation of OF tip

The polymeric cladding was removed with the aid of a blade and then wiped with a cloth soaked in ethanol to remove the cladding remnants. **Figure S1 (a)** shows the very smooth surface of an uncoated fiber tip, and by EDX analysis (**Figure S1 (b)**), only the elements silicon (Si) and oxygen (O) were identified, as the core is made of SiO_2. This procedure should be gently performed with careful attention to be sure that all the cladding is removed. For instance, **Figure S2** displays a gold-coated tip with remnant cladding, which has a negative impact on the fiber sensitivity. The presence of the cladding (a hard fluorinated polymer), between the core and the gold film, was confirmed by EDX analysis, where fluorine (F) was identified in a layer with a thickness of around 10 μm.

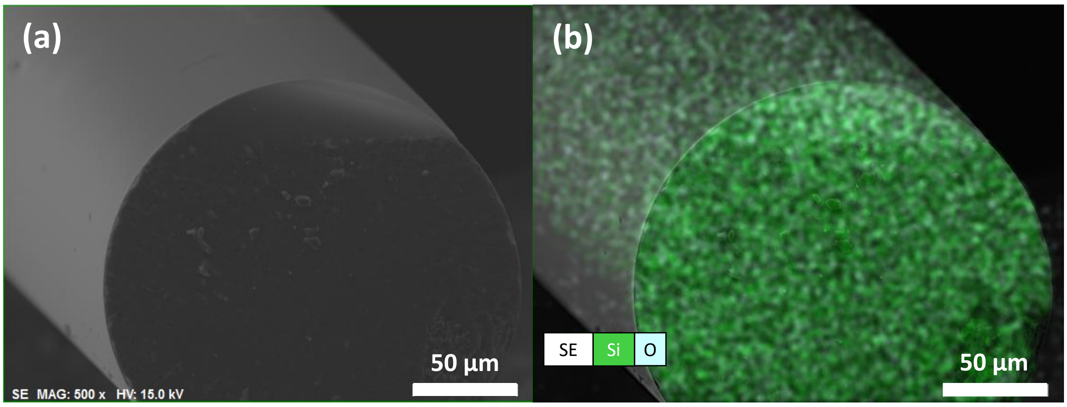

Figure S1. (a) SEM image and (b) EDX mapping of an uncladded fiber tip.

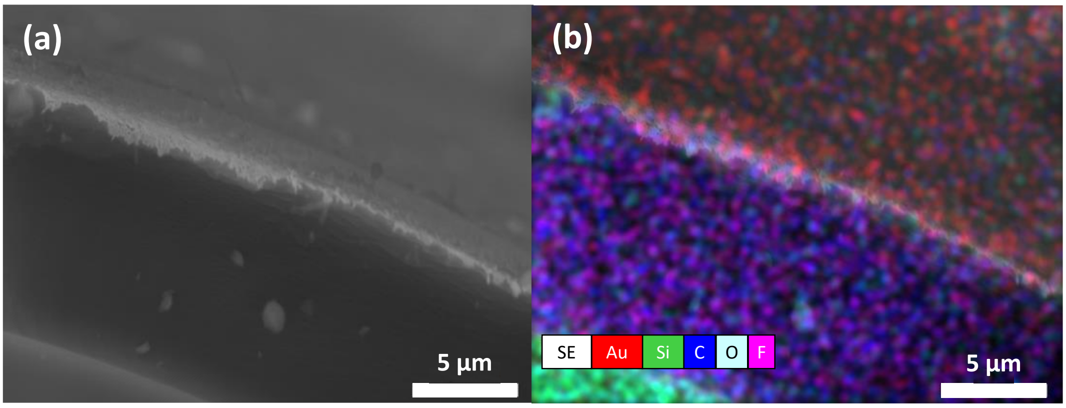

Figure S2. (a) SEM image and (b) EDX mapping of the cross-section of a gold-coated tip containing remanent cladding around the core.

S1.2 Gold coating on OF tip

Uncladded OF tips were coated with a thin-film of gold by the sputtering technique. Then, the tips were annealed in a thermal chamber, at 180 °C for 2 hours, to enhance the adhesion of the gold film on the silica surface of the tips. In **Figure S3** (a), it is possible to observe the surface of a non-annealed tip, presenting a homogeneous layer of gold. With the magnification of the tip surface in **Figure S3** (b), the presence of small grains is observed.

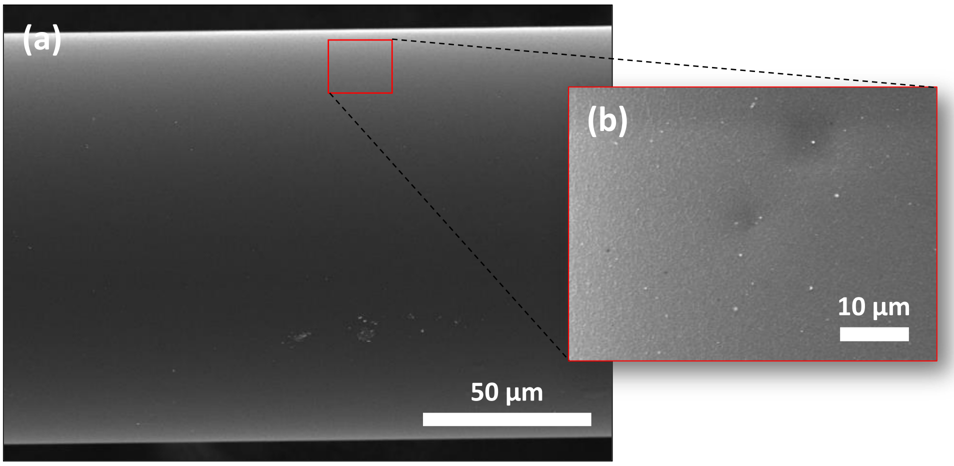

Figure S3. SEM images of a gold-coated 200 µm tip before the thermal annealing treatment.

Figure S4 (a) shows the surface of a tip after thermal annealing. In **Figure S4** (b), it is possible to observe that the standard morphology of the gold film with the presence of isolated gold grains changed to an interconnected film without any percolation, making the film more homogeneous, due to the aggregation of gold grains. Finally, in **Figure S4** (c), the thickness of the gold layer after annealing was measured, being around 56 nm.

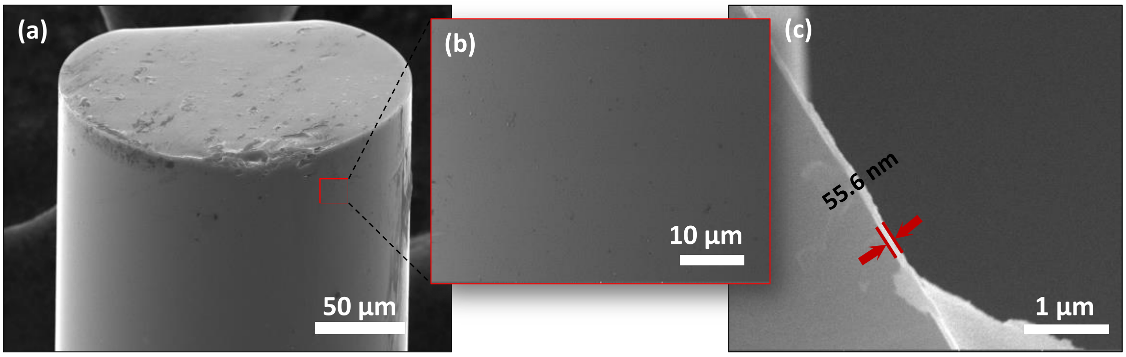

Figure S4. SEM images of a gold-coated 200 µm tip after the thermal annealing treatment. In (c), the detail of the gold film thickness is observed.

An SEM-EDX characterization was also performed on the gold-coated tips (after annealing), revealing the presence of the Au well-distributed on the SiO_2 core surface.

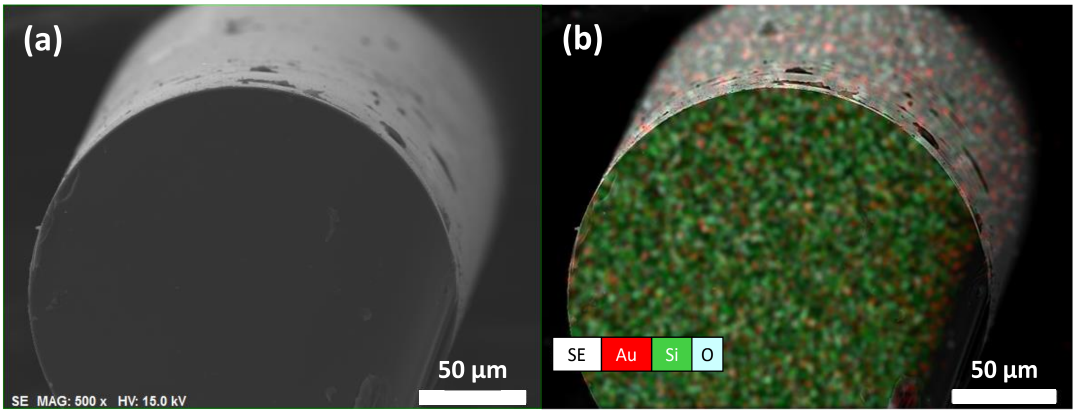

Figure S5. (a) SEM image and (b) EDX mapping of gold-coated uncladded tip.

S1.3 Biofunctionalization of a gold-coated OF tip

The biofunctionalized gold-coated tips were characterized by SEM-EDX and also by AFM to evaluate the surface topography after the biofunctionalization steps.

The results obtained for the tip after being biofunctionalized also indicate a uniform and homogeneous gold film, as illustrated in **Figure S6**. Moreover, the SEM-EDX mapping image shows the presence of carbon (C) on the gold coating, which comes from the biofunctionalized layer. The biofunctionalization with ABs and tested with NT-proBNP seemed to not affect the previously deposited gold film, as expected.

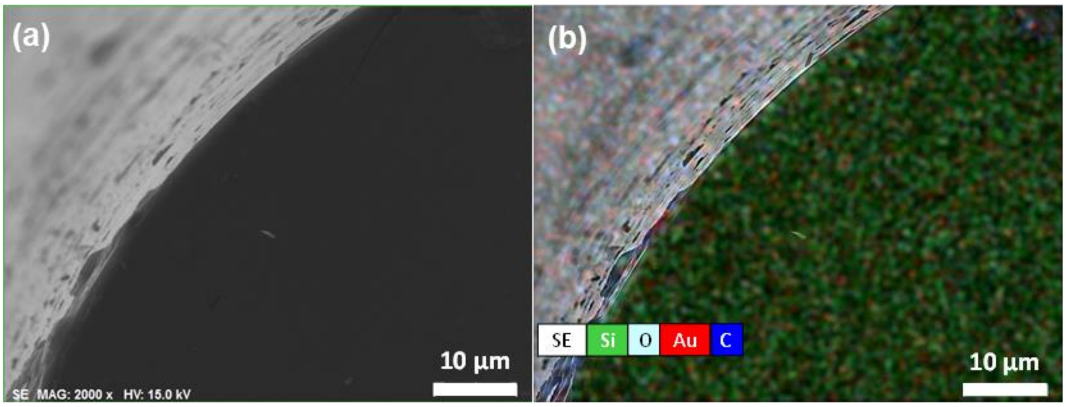

Figure S6. (a) SEM image and (b) EDX mapping of a tip after the biofunctionalization and NT-proBNP detection tests.

The topographies of bare and of biofunctionalized annealed tips were analyzed by AFM. The roughness parameter was evaluated based on measurements performed in areas of 1 μm^2 from different locations of the tip surface. In **Figure S7**, it is possible to observe 3D images from the surface of the bare and the biofunctionalized tips, where there is a greater homogeneity of the surface of the latter.

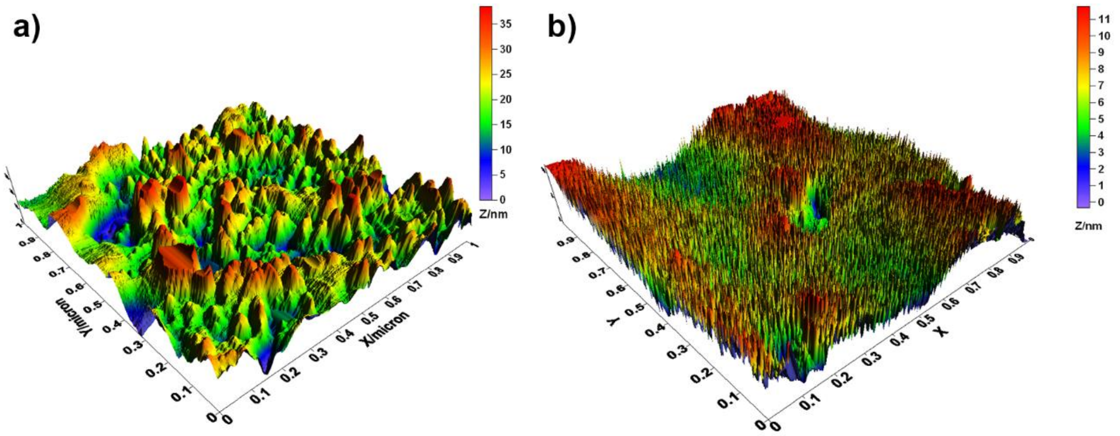

Figure S7. Contact-mode AFM 3D topography images made on (a) bare and (b) biofunctionalized 200 μm tips. Scan areas: 1 μm^2.

After the biofunctionalization step, the analyzed surface exhibited a roughness with a substantially reduced standard deviation, from about 35 nm to 11 nm, as expected and reported in [68]. The higher roughness shown in Figure S7 (a) for the bare tip can be explained by the surface heterogeneity induced by the overlapping of gold composed of isolated gold grains deposited by sputtering, even after annealing to homogenize the surface and turning the grains into an interconnected film. Overall, generating rough surfaces is one of the most convenient and effective ways to improve sensing signals. After the gold substrate modification step with the functionalization of anti-NT-proBNP ABs and passivation with BSA, the roughness decreased, suggesting a densely packed and homogeneous modified surface. This is due to the formation of a self-assembly monolayer of cysteamine to which AB molecules are immobilized through amide coupling via NHS/EDC chemistry. The addition of BSA during the functionalization would still have improved the surface coverage, due to the adsorption of BSA molecules at different angles.
